# Prevention and Management of Complications in Laparoscopic Myomectomy

**DOI:** 10.1155/2018/8250952

**Published:** 2018-03-05

**Authors:** V. Tanos, K. E. Berry, M. Frist, R. Campo, R. L. DeWilde

**Affiliations:** ^1^University of Nicosia Medical School, Nicosia, Cyprus; ^2^European Academy for Gynecological Surgery (Nicosia Branch), 55-57 Andrea Avraamidi St., Strovolos, 2024 Nicosia, Cyprus; ^3^Aretaeio Hospital, Strovolos, Nicosia, Cyprus; ^4^St George's, University of London MBBS Programme at the University of Nicosia Medical School, Nicosia, Cyprus; ^5^European Society Gynaecological Endoscopy, Leuven, Belgium; ^6^The European Academy for Gynecological Surgery, Leuven, Belgium; ^7^Life Expert Centre, Schipvaartstraat 4, 3000 Leuven, Belgium; ^8^Cahir Clinic of Gynecology, Obstetrics and Gynecological Oncology, University Hospital for Gynecology, Pius-Hospital Oldenburg, Medical Campus University of Oldenburg, Oldenburg, Germany

## Abstract

Myomectomy aims to preserve fertility, treat abnormal uterine bleeding, and alleviate pain. It should cause minimal damage to the endometrium, while being tolerable and durable, and reduce the incidence of myoma recurrence and complications including bleeding, hematoma, adhesions, and gravid uterus perforation. Training and experience are crucial to reduce complications. The surgical strategy depends on imaging information on the myomas. The position of the optical and secondary ports will determine the degree of ergonomic surgery performance, time and difficulty of myoma enucleation, and the suturing quality. Appropriate hysterotomy length relative to myoma size can decrease bleeding, coagulation, and suturing times. Bipolar coagulation of large vessels, while avoiding carbonization and myometrium gaps after suturing, may decrease the risk of myometrial hematoma. Quality surgery and the use of antiadhesive barriers may reduce the risk of postoperative adhesions. Slow rotation of the beveled morcellator and good control of the bag could reduce de novo myoma and endometriosis. Low intra-abdominal CO_2_ pressure may reduce the risk of benign and malignant cell dissemination. The benefits a patient gains from laparoscopic myomectomy are greater than the complication risks of laparoscopic morcellation. Recent publications on laparoscopic myomectomies demonstrate reduced hospitalization stays, postoperative pain, blood loss, and recovery compared to open surgery.

## 1. Introduction

Uterine fibroids are a common disorder with an estimated incidence of 20–40% in women during their reproductive years [1, 2]. Myoma diagnosis has been substantially improved in the last decade, mainly due to higher sensitivity and specificity of imaging modalities and improved knowledge about how a myoma alters normal endometrial function. The frequency of myoma varies according to age, inheritance, and possibly body mass index [3]. The fact that more women are seeking childbirth at a later age increases the frequency of infertility due to myoma presence, and reduces the implantation potential. Submucous and intracavitary myomas are usually operated on using hysteroscopy, while subserous myomas are approached with laparoscopy. In comparison, intramural myomas can be operated by hysteroscopy or laparoscopy depending on the size (<4 cm) and surgeon's experience. The number, size, location, and vascularization of a myoma as well as the experience of the surgeon predict the outcome of the operation and subsequent risk of complications [3].

Recent studies demonstrate that the complications after myomectomy have been increasing in the last decade [4, 5]. This trend can partially be attributed to the shift toward childbearing at a later age. Problems with infertility, as well as progressively larger myomas that are also increasing in number, are more common in this age group. A growing number of gynecologists with unjustified confidence at myoma excision by minimally invasive surgery (MIS) without sufficient training in laparoscopic suturing and electromechanical morcellation might also be attributed to these statistics [6]. In 2002 a meta-analysis reported a similar number of intra- and postoperative complications after gynecological operation performed by laparotomy or by laparoscopy [7]. Another meta-analysis on 6 RCTs with 576 patients comparing laparoscopic versus open myomectomy, demonstrated that laparoscopic myomectomy was associated with faster postoperative recovery by day 15, reduced operative blood loss, diminished postoperative pain, and fewer overall complications. Authors concluded that laparoscopic myomectomy, performed by a specialized surgeon and with more stringently selected patients, is a better choice than open surgery [8].

Injuries may be direct, such as by organ damage due to morcellation. Alternatively, an indirect injury could occur while performing MIS on a sarcomatous tissue of a presumed myoma. This would result in the spreading of malignant cells, causing peritoneal parasitic myoma or endometriosis. Complications might arise due to incorrect selection of the patients, failure to limit time in laparoconversion, and surgeons' overconfidence in managing difficult cases [9, 10]. The intraoperative and postoperative laparoscopic myomectomy complications as well as preventive measures are reviewed below.

## 2. Methods

A systematic review on published studies in PubMed, EMBASE, CBMdisc, Ovid, and Cochrane, with cross-referencing, was performed. Myomectomy after laparoscopy, hysteroscopy, and complications were the main key words used to isolate the relevant articles.

### 2.1. General Considerations

Laparoscopic myomectomy complications can be intraoperative due to inappropriate hysterotomy, enucleation, hemostasis, and morcellation injuries. Alternatively, the complications may be postoperative due to hysterotomy site hematoma and adhesions, pelvic adhesions, and recurrence. Obstetric complications after laparoscopic myomectomy are also possible. Laparoscopic resection of 654 fibroids, with a mean myoma size of 5.3 cm, had an intraoperative complication rate of 2.6% and postoperative complications occurred in 5.7% of patients [4]. In another study, of 2,050 laparoscopic myomectomies, the total complication rate was 11.1% (225/2050 cases) [11].

In another study, laparoscopic myomectomy was performed in 505 women, removing 912 myomas, and 184 (36.4%) patients had multiple myomectomies. A comparison between the size of the myomas (<10 cm and ≥10 cm in largest diameter) and number of myomas removed (≤4 and ≥5 myomas) was performed. The mean blood loss, duration of surgery, and hospital stay were greater in the groups with multiple and larger myomas [12]. An odds ratio was computed to estimate the risk of complications in relation to the patient characteristics which showed that the probability of complications significantly rises with an increase in number of myomas (more than 3 myomas OR: 4.46, *p* < .001) and with either intramural (OR: 1.48, *p* < .05) or intraligamentous location of myomas (OR: 2.36, *p* < .01), whereas the myoma size seems to primarily influence the risk of major complications (OR: 6.88, *p* < .001) [13].

Authors in the above studies concluded that laparoscopic myomectomy, when performed by an experienced surgeon, can be considered a safe technique with an extremely low failure rate, low complication rate, and good results in terms of subsequent pregnancy outcomes [11–13].  Table 1 demonstrates that the risk of longer operation time, blood loss, blood transfusion, and hysterectomy hematoma is higher with increasing size of the myoma. Furthermore, a double-blind study on pain control comparing 19 cases after laparoscopic myomectomy and 21 after laparotomy showed that laparoscopic surgery had clear advantages over laparotomy as far as pain control is concerned [13].

### 2.2. Intraoperative Complications

Excessive blood loss, myometrial hematoma, and morcellation accidents are the most frequent intraoperative complications during laparoscopic myomectomy. The risk factors contributing to intraoperative bleeding are insufficient use of vasoconstrictive agents, fibroid size, fibroid position, number of fibroids, failure to identify the cleavage plane, failure to identify the feeding vessel, insufficient hemostasis, lack of precision in suturing, loose knotting, and surgeon inexperience (Table 3) [10]. Uterine wound approximation problems present as the most frequent intraoperative complications when adenomyosis/adenomyoma is present.

In hysterotomy, the incision is performed along the trocar projection line and is used for the needle holder and direction of suturing. The incision length depends on the fibroid size, how much it bulges, and its orientation. The size of the incision should be similar to the length of the myoma. The selection of the incision area, orientation, and length will determine the degree of difficulty of myoma enucleation, suturing, and amount of bleeding. The reported usual volume of bleeding ranges from 84 to 1200 mL according to several papers [15–18]. The excessive bleeding and failure to control it are also the major reasons for conversion to laparotomy, which has been reported between 0.34% and 2.7% [9, 11, 18, 19]. These authors agree that, increasing the number and size of the fibroids, the hemorrhage risk of bleeding is increasing. Complications due to incomplete myoma excision create the possibility of continuous bleeding over several weeks, intermittent bleeding to menometrorrhagia, single pain episodes, severely painful dysuria, chronic cystitis, constipation, and bowel spasms. Very seldom peritonitis occurs, and there is a higher recurrence rate in these cases [10].

Very large myomas have fewer reports. Laparoscopic Excision has been reported in 51 women with at least one myoma larger than 9 cm. Overall 78 myomas were operated with a mean operating time of 136.67 ± 38.28 minutes (range 80–270 min) and mean blood loss 322.16 ± 328.2 mL (range 100–2000 mL). One patient developed a broad ligament hematoma, two developed postoperative fever, and one underwent open subtotal hysterectomy 9 hours after surgery for dilutional coagulopathy. Authors concluded that myomectomy for very large fibroids by laparoscopy is a safe alternative to laparotomy for very large myomas [20].

### 2.3. Postoperative and Long-Term Myomectomy Complications

Intra-abdominal and intrauterine adhesions are delayed postmyomectomy complications. The use of antiadhesive agents might be helpful for reducing postoperative adhesions; however, strong evidence for this in the literature is missing [10]. A prospective observational study, performing hysteroscopy in 51 infertile patients three months after myomectomy, reported intrauterine adhesions (IUA) in 11 out of 51 (21.57%) cases. No significant relationship was found between IUA and the type, size, or number of fibroids. Additionally, no relationship was found between use of IUA and endometrial trauma during the myomectomy. Postoperative hysteroscopy is highly recommended to diagnose and treat these adhesions early [20].

When concomitant pathologies exist such as focal adenomyosis and adenomyoma, or in large intramural and submucous myoma extractions, myometrium approximation becomes a big challenge. Sometimes a gap left between the wound edges cannot be avoided. Accordingly, a postoperative follow-up is recommended and a comment in patient's discharge report is advisable. Special attention should be paid to these patients, especially if the decision is made to pursue spontaneous delivery.

Obstetric complications due to uterine perforation during labor are caused mainly due to weak myometrium after destruction by extensive coagulation and/or myometrial tissue injury, or after defective suturing and poor tissue approximation. Preoperative assessment of submucosal fibroids is essential for the decision on the best approach for treatment. In the infertile population, cumulative pregnancy rates by the laparoscopic and the minilaparotomy approaches are similar, but the laparoscopic approach is associated with a quicker recovery, less postoperative pain, and less febrile morbidity [3].

Evaluating the obstetric complications, the pregnancy rate in term infants was 57.1% after myomectomy without any uterine rupture as reported by Altgassen et al. [4]. Among patients wanting pregnancy after myomectomy, the pregnancy rate of 69.8% was noted and only one (0.26%) recorded a spontaneous uterine rupture at 33 weeks' gestation [21]. In another series of 1,032 laparoscopic myomectomies there were only 6 obstetric complications [10]. Out of 130 patients desiring pregnancies, 78 (60%) became pregnant. Among the 78 pregnancies, there were 6 abortions, 60 spontaneous deliveries, and 18 caesarean sections. Eight sets of twins and one set of triplets were reported [10]. The reported uterine rupture rate in 3rd trimester pregnancies and women in labor is quite small and the major reason leading to this complication is myometrium insufficient healing [22].

### 2.4. Morcellation Complications

The exact incidence of morcellation complications is unknown and likely underestimated. Medical literature mainly describes case reports and the vast majority of complications after tissue power morcellation are not reported.

#### 2.4.1. Direct Injury

A systematic review of surgical centers performed in the United States from 1993 to 2013 registered 55 morcellator-related complications. Organs injured included large bowel (*n* = 31), vascular system (*n* = 27), the kidneys (*n* = 3), ureters (*n* = 3), the bladder (*n* = 1), and the diaphragm (*n* = 1). Significantly, in six cases, it was reported that patient death followed. The complications were noted intraoperatively in most patients (66%); however in the remainder of cases they were not identified for up to 10 days after operation. This finding was attributed to surgeon inexperience [5]. It has been reported that the overall probability of direct power morcellator injuries to internal organs is more frequent (0.12%) than morcellator injuries to the abdominal and pelvic wall (0.06%). A survey of ESGE physicians found that most had not experienced bladder, ureter, or aorta and vessel injuries during morcellation. However, three surgeons with morcellator experience did report causing permanent damage. In contrast to the findings of M. P. Milad and E. A. Milad, no death was reported. Morcellator technical problems were also found to be a rare issue (0.12–0.3%), with transient stacking being the most frequent issue. Ultimately, direct morcellator injury is a reportedly rare occurrence. Despite the low risks, the ESGE board maintains that only physicians with adequate training and knowledge should be performing these endoscopic procedures in order to avoid these rare, but serious complications [27].

#### 2.4.2. Indirect Injury

The overall incidence of parasitic fibroids after laparoscopic surgery in general with the use of morcellation was reported to be between 0.9% and 0.12% [24, 28, 29]. The reported incidence of parasitic myomas after laparoscopic myomectomy was found to be 0.2–1.2% [28, 29] (Table 2). In a survey study the risk for parasitic myoma after myomectomy was reported as 0.08% [24], much lower than 0.12–0.95% found after a meta-analysis of 44 studies [28].

Posthysterectomy pelvic adenomyotic masses were observed in 8 out of 1,405 (0.65%) after laparoscopic subtotal hysterectomies [35]. In a case control study after 277 laparoscopic supracervical hysterectomy (LASH) with uterine morcellation compared with 187 patients after VH or TAH (no morcellation), the de novo diagnosed endometriosis was 1.4% (3/217) in the LASH group and 1.4% (2/145) in the control subjects. The risk of de novo formation of endometriosis after adenomyoma morcellation is much higher 0.16% and may be attributed to the fact that many endometriosis cells are already buried below the peritoneal epithelium while immunological factors might also be involved in favor of dissemination and growth [24]. In addition, 25% of the enlarged uteri with myomas might also concomitantly include adenomyosis [23, 26].

The risk of parasitic myoma and de novo endometriosis after laparoscopic myomectomy might be also explained by the fact that this operation is performed more often in premenopausal women with higher estrogen levels [23, 35]. Postmenopausal women treated with HRT have a higher risk of de novo tissue complication; hence patients should be informed accordingly prior to the operation. The time of exposure to morcellation process, larger tissue volume to be morcellated, greater amounts of fragments released, and the higher CO_2_ intra-abdominal pressure needed may all contribute to rising the risk of de novo formation of uterine tissue implantation. Stabilization of the specimen prevents fast rotation and spread of cells and tissue fragments in the abdominal cavity while in-bag morcellation is another option, although it is still under investigation. Efforts should be made to remove all tissue fragments after morcellation and use thorough irrigation and suction in the peritoneal cavity.

The survival rate of patients with leiomyosarcoma operated on by laparoscopy with myoma morcellation has been reported to be decreased, concluding that primary surgery involving tumor injury seems to be associated with a worse prognosis [36–38]. The benefits of myomectomy should be weighed against the risks, and management of fibroids in perimenopausal women should be individualized. Whether power morcellation poses a unique danger to the patient with occult LMS is still an unanswered question [38]. Patients over the age of 40 with myomas >8 cm in diameter, with hypervascularity and necrosis recorded in ultrasound and MRI are considered highly suspicious for leiomyosarcoma [37]. Rossetti et al., in their review published in April 2001, looked at the rate of myoma recurrence following either laparoscopic or laparotomic myomectomy (162 patients, 82 for each type of surgery) [39]. Patient follow-up continued for up to 40 months. At the end of this time, 11 in the laparoscopy and 9 in the laparotomy group had suffered a recurrence. Analysis did not show any statistical significance [39]. The higher the number of fibroids and the larger the size of the myoma, the higher the risk of recurrence [40].

#### 2.4.3. Prevention of Late Complications of Morcellation

To minimize the risk of upstaging uterine sarcomas and benign tissues such as fibroid and endometriosis tissue, power morcellation of a presumed fibroid can be performed in a laparoscopic bag. Research in tissue retrieval from the abdominal cavity mainly focuses on in-bag tumor morcellation. Evidence suggests that in-bag tumor morcellation may prevent parasitic fibroids, reduce the risk of upstaging premalignant lesions, and offer protection from direct morcellation trauma [41–43]. In urology, in-bag morcellation after laparoscopic removal of early stage and low-grade renal cell carcinoma is reported to be safe and effective. Of course, in aggressive manipulations or especially in difficult cases the laparoscopic bags can be torn. In these instances, spillage of tumor cells can occur. The use of methylene blue dye in the lap-bag has been suggested in order to create awareness once spillage has occurred. Transvaginal in-bag morcellation has also been described; however further prospective and well-designed studies are needed before establishing the potential value of in-bag morcellation in gynecologic surgery [21, 42–44].

### 2.5. Strategies to Prevent Possible Surgical Complications

#### 2.5.1. Imaging and Evaluation of Fibroids for Malignant Changes

The anatomic location and the features of a myoma are important parameters for planning the operation. The surgery outcome and the risk of intraoperative complications are highly dependent on trocar placement, finding of the correct cleavage plane, hemostasis, and suturing technique. Preoperative differentiation between myoma, adenomyoma, and LMS of vital importance will define the way of surgery. Ultrasonographic diagnosis is the primary and most effective screening tool to investigate fibroid size, number, and location with high precision [45]. For cases that need further investigation regarding the fibroids proximity to adjacent organs, or that are suspicious of being a sarcoma, MRI is recommended to follow. The MRI can provide more details, such as information about increased vascularity and necrosis. Where multiple fibroids with complex morphology are found, the surgeon should be highly suspicious of a sarcoma [46]. The reproducibility of MRI is higher than US regarding the very big size and number of fibroids.

Occasionally high contrast sonography and/or hysteroscopy can evaluate the uterine cavity involvement and surgeon might suggest GnRHa prior to surgery. This is important for infertility treatments, where conservative myomectomy is crucial in determining future embryo implantation and term pregnancy potential. The diagnosis of a big intramural myoma, involving also the junctional zone of the endometrium, is crucial to be noted by the surgeon prior to the operation. Also methylene blue dye can be injected intracavitary, to easily recognize the entry into the endometrial cavity during surgery [47]. The development of new technologies in diagnostic procedures like 4D and hystero-contrast-sonography, MRI, and ambulatory hysteroscopy has assisted in early diagnosis and accurate follow-up.

There are no imaging techniques that can demonstrate any characteristic features for leiomyosarcoma (LMS) [48–50]. Ultrasound comparison between eight LMS and three STUMPs with 225 fibroids demonstrated that LMS were significantly larger, solid tumors with diameter ≥8 cm, than other uterine smooth muscle tumors [51]. However, rapid increase in size (within 3 months) is generally not distinctive as it may occur in benign fibroids as well [52–54]. High central vascularity, combined with other sonographic findings, increases the positive predictive value to 60%, but sensitivity decreases to 75%. Detecting sarcomas by 2D US and Power Doppler (USPD) findings mainly depends on the nature of the tumor. The peak systolic velocity sensitivity is about 80% and the specificity 97%. No studies on sarcoma diagnosis have been published on vascular indices measured by 3D USPD. Degenerative cystic changes can be also observed as well with an increased peripheral and central vascularity. LMS have a similar appearance to fibroids on US and MRI [49, 52]. However, a large >8 cm, solitary, oval shaped, highly vascularized and irregular, heterogeneous myometrial tumor with central necrosis/degenerative cystic changes and absence of calcifications should raise suspicion of a LMS [48, 51, 55, 56].

### 2.6. Preventive Measures

#### 2.6.1. Medical Management Facilitating Laparoscopic Myomectomy, Reduction of Myoma Size

Systemic hormone therapy and contraceptives are generally the first line of treatment, especially for patients with heavy menstrual bleeding. Nonhormonal treatment options are also available, such as tranexamic acid and nonsteroidal anti-inflammatory agents [57]. Another option is the use of Gonadotropin-releasing hormone (GnRH) or ulipristal acetate as a preoperative measure for large myomas in order to reduce the risk of hemorrhage [58–60]. Usually with treatment for 6–8 weeks, fibroids shrink by 30–50%. The use of these agents should not exceed the 6–8 weeks, as they can obscure the tissue planes between fibroids and the myometrium, making enucleation difficult and increasing the chance of myoma recurrence [61]. Once this agent is discontinued, myoma regrowth occurs; hence the timing of the operation close to the end of the treatment is necessary. Both the cost of the medication and the risk of cleavage plane lost must be weighed against the benefits when deciding whether or not to use them.

#### 2.6.2. Management of Anemic Patients

Resolution of preoperative anemia can be achieved by a number of ways, including intravenous or oral administration of iron supplement and folic acid, administration of oral contraceptive pills, or administration of another hormonal medication used to stop or decrease severe menstrual bleeding. As previously mentioned GnRHa treatment can be used preoperatively to reduce myoma size and vascularization and thereby prevent hemorrhage. Drugs that modulate progesterone action, such as ulipristal acetate, may also decrease symptoms and shrink fibroids. Ulipristal acetate is approved for three months of therapy prior to myomectomy. Treatment with ulipristal acetate also can achieve substantial volume reduction and cease metrorrhagia [62, 63].

A systematic review and meta-analysis of randomized controlled trials on GnRHa administration prior to laparoscopic myomectomy reported 3 studies with 168 patients. Pretreatment with GnRHa did not reduce operative time, but a significant reduction of the intraoperative blood loss was noted. Statistical difference was also observed in postoperative hemoglobin concentration (mean difference, 1.15 g/dL; 95% CI, 0.46–1.83) and red blood cell count (mean difference, 0.65 × 10^6^ cells/mL; 95% CI, 0.16–1.14) but not serum iron concentration. None of the patients in the studies experienced any major intraoperative or postoperative complications, and only 1 patient in each group required blood transfusion [64].

#### 2.6.3. Prevention of Adhesion Formation

The risk of adhesion formation on hysterotomy site and pelvis after laparoscopic myomectomy has been reported up to 92% and 76%, respectively [65–67]. Use of antiadhesive barriers may reduce the risk of postoperative adhesion formation. However, good surgery with respect to minimal destruction and handling of the healthy tissue, avoiding unnecessary organ manipulation, controlled bleeding, minimal coagulation, and reasonable operating time remain the best ways to diminish the risk of adhesion formation [68]. Physical barriers, oxidized regenerated cellulose, icodextrin, and other materials are all used to cover the myomectomy wound as a preventive measure against adhesion formation [69]. There is no conclusive evidence on the relative effectiveness of these interventions. Low quality evidence suggests that oxidized regenerated cellulose (Interceed), expanded polytetrafluoroethylene (Gore-Tex), and sodium hyaluronate with carboxymethylcellulose (Seprafilm) may all be more effective than no treatment in reducing the incidence of adhesion formation following pelvic surgery including myomectomy [70].

## 3. Discussion

Control of bleeding is of paramount importance after myoma enucleation. Bipolar diathermy is preferable to monopolar diathermy, as it targets only the big vessels and causes less destruction to healthy myometrium. Hemostasis should be avoided for micro bleeders in order to facilitate healing process. Excessive coagulation and carbonization should be avoided since it can be detrimental to myometrial healing. Using bipolar diathermy, a dissecting grasper, and a suction cannula, meticulous exploration of the dissection field can more efficiently detect and coagulate any actively bleeding vessels. Injection of ADH (vasopressin derivative solutions) or diluted adrenalin around the fibroid wall (extracapsular) causes blood vessels to constrict and minimizes the bleeding to facilitate dissection. Similarly, temporary bilateral uterine artery clipping can reduce blood supply and bleeding during myoma excision [71]. Additionally, the use of preoperative treatment to improve the hemoglobin concentration and diminish myoma size primarily applies to infertility cases where the myomas deform the cornua and the endometrial cavity.

Appropriate use of imaging and planning is of high importance. These tasks will help with the positioning of the optical and secondary ports, which greatly define the degree of ergonomic surgery performance, the amount of time required, and the difficulty of myoma enucleation and suturing. The use of antiadhesive barriers may reduce the risk of postoperative adhesion formation as well. However, good surgery with respect to minimal destruction and handling of the healthy tissue, avoiding unnecessary organ manipulation, controlled bleeding, minimal coagulation, and reasonable operating time remain the best ways to diminish the risk of adhesion formation [68].

Parasitic myomas are a known risk of laparoscopic myomectomies. Although they occur rarely, in up to 0.9% of cases, they should be avoided. Ongoing research suggests that morcellation in open bag, with slow blade rotation and controlled by beveled longer tip placed anteriorly, can probably reduce cell dissemination of parasitic myoma and endometriosis. Additionally, appropriate intra-abdominal CO_2_ pressure may also reduce the risk of cell dissemination [41].

Even with the use of in-bag morcellation, these devices should not be applied to remove suspected malignant tissues. Low-risk patients should be adequately informed preoperatively that morcellation can spread cancer cells in the unlikely case of hidden malignancy and not be falsely assured that uterine masses are not cancerous. An objective explanation that there is no way to completely exclude cancerous cells within the myometrium or in a myoma should be offered [36, 37].

Training and experience are crucial in reduction of laparoscopic myomectomy complications. Knowledge about assembly and use of the morcellator can help to avoid device technical accidents and direct injuries. Direct visualization by placing the device, maintenance of pneumoperitoneum, careful placement of the morcellation blade as previously described, and handing the to the morcellation tenaculum rather than moving the tenaculum to the specimen can all minimize power morcellation accidents.

The patient's safety in laparoscopy depends upon patient selection, surgeon's training and skills, equipment fidelity, instrument reliability, and hospital directives and policies. The patient selection depends directly on surgeon's capabilities and indirectly on hospital directives. Recent evidence based studies have shown that certain exercises and training can improve both novices' and experts' skills far more than the traditional apprentice–student method [72]. The patients' safety after laparoscopic myomectomy is preserved, presenting excellent treatment results with short hospitalization stays, immediate mobilization, and reduced postoperative pain.

## Figures and Tables

**Table 1:** Comparison of hysterotomy scar hematoma after laparoscopic myomectomy.

Study	Patients	Myoma number	Myoma mean size cm	Operation average time min	Blood loss	Blood transfusion	US Hysterotomy scar hematoma
Sinha et al., 2003 [14]	51	78	>9	137	323 ml	1 (1.3%)	1 (1.3%)
Altgassen et al., 2006 [4]	351	654	5.3	113	ND	1	(29.2%)
Sizzi et al., 2007 [11]	2050	ND	6.4	ND	14 (0.68%) hemorrhage	3 (0.14%)	10 (0.48%)
Sinha et al., 2008 [12]	505	912	5.9	60	90 ml	ND	ND
Mettler et al., 2012 [10]	335	480	4–9	90	157 ml	0	21 (6.2%)

*Note*. ND: no data.

**Table 2:** De novo myoma and endometriosis formation after laparoscopic myomectomy/hysterectomy.

Study	Type of study	Study details	Parasitic myoma%	Parasitic endometriosis%
Tanos et al., 2016 [23]	EGSE Survey	191 doctors participated	0.08	0.16
Meulen et al., 2016 [24]	Meta-analysis 44 studies	Laparoscopic morcellation and myomectomy	0.12–0.95 0.2–1.2	ND
Schuster et al., 2012 [25]	Case control	277 LASH morcellations, 187 VH or TAH	ND	1.4 1.4
Donnez et al., 2007 [26]	Retrospective	8 out of 1405 LASH cases	ND	0.65

*Note*. ND: no data, LASH: laparoscopically assisted subtotal hysterectomy, VH: vaginal hysterectomy, and TAH: total abdominal hysterectomy.

**Table 3:** Intraoperative bleeding during laparoscopic myomectomy without any vasoconstrive measures.

Study	Myoma type	Patients number	Myoma size cm	Myoma multiple	Bleeding ml	Hospitalization	Conversion to LMY
Sizzi et al., 2007 [11]	ND	2050	>4	48	ND	ND	0.34
Tinelli et al., 2012 [15]	SS + IM	235	4–10	48	118 +/− 28	86% 48 hs	0
Malzoni et al., 2006 [30]	IM -75%	982	6.7 +/− 2.7	47	3/982	ND	1.29
Sankaran and Odejinmi, 2013 [31]	ND	125	7.6	3.7	ND	ND	1.6
Dubuisson et al., 1995 [32]	IM	71	>5	ND	ND	ND	2.7
Saccardi et al., 2014 [33]	ND	444	8–12	ND	2/444	ND	1.35
Walid and Heaton, 2011 [16]	ND	41	2–15.6	ND	2–1200 ml	ND	ND
Mallick and Odejinmi, 2017 [19]	IM 49% SS 33%	323	7.7 +/− 2.8	4 +/− 3.6	279 +/− 221	1.9 +/− 0.95 days	0.62
diZerega, 1997 [18]	IM 34% SS 19%	54	>3	ND	84	2.09 days	1.8
Mathew et al., 2013 [34]	ND	1,001	ND	44	248 mL avg, 1 transf	1–5	1 death pop unexp

*Note*. ND: no data.
